# Crystal-like order and defects in metazoan epithelia with spherical geometry

**DOI:** 10.1038/s41598-020-64598-w

**Published:** 2020-05-06

**Authors:** Daria S. Roshal, Karim Azzag, Emilie Le Goff, Sergei B. Rochal, Stephen Baghdiguian

**Affiliations:** 1 Faculty of Physics, Southern Federal University, Zorge 5, Rostov-on-Don, 344090 Russian Federation; 2 ISEM, Univ Montpellier, CNRS, EPHE, IRD, Montpellier, France; 3 Lillehei Heart Institute, Department of Medicine, University of Minnesota, Minneapolis, MN USA

**Keywords:** Surfaces, interfaces and thin films, Biomaterials - cells, Biological physics

## Abstract

Since Robert Hooke studied cork cell patterns in 1665, scientists have been puzzled by why cells form such ordered structures. The laws underlying this type of organization are universal, and we study them comparing the living and non-living two-dimensional systems self-organizing at the spherical surface. Such-type physical systems often possess trigonal order with specific elongated defects, scars and pleats, where the 5-valence and 7-valence vertices alternate. In spite of the fact that the same physical and topological rules are involved in the structural organization of biological systems, such topological defects were never reported in epithelia. We have discovered them in the follicular spherical epithelium of ascidians that are emerging models in developmental biology. Surprisingly, the considered defects appear in the epithelium even when the number of cells in it is significantly less than the previously known threshold value. We explain this result by differences in the cell sizes and check our hypothesis considering the self-assembly of different random size particles on the spherical surface. Scars, pleats and other complex defects found in ascidian samples can play an unexpected and decisive role in the permanent renewal and reorganization of epithelia, which forms or lines many tissues and organs in metazoans.

## Introduction

As well known, genetic control is the basic principle in development and homeostasis of metazoans^1^. However, like viruses and supramolecular structures, such as DNA^2–6^, single-layer packings of cells forming epithelial monolayers obey simple physical and topological principles controlling their structural organization^7^. Study of these principles for epithelial monolayers is very important since the simplest cellular packings contributes significantly to the maintenance of tightness during epithelial morphogenetic reorganizations, embryonic development and the permanent renewal of some tissues and organs throughout metazoan adult life. In this context, numerous studies were focused on the crucial role of molecular motors, genetic induction and junctional complexes^8–10^. In addition, the epithelial monolayers represent a convenient testing ground for current biological investigations of apoptosis, necrosis and cell division processes^11–13^.

The vast majority of epithelial monolayers are proliferative and thus dynamic^14^, which naturally complicates the investigation of physical rules governing their sub-macroscopic structural features. However, in living systems there are some non-proliferative exceptions as is the case for the *Drosophila* Malpighian tubules epithelial monolayer^15,16^ or the spherical epithelial monolayers (SphEMs) that constitute the follicular system covering the eggs of ascidians^7,17^. In most ascidians, the follicular system is organized in layers. The external layer is a shell of follicular polygonal cells adhering to an extracellular matrix, known as the chorion. The next layer contains so called test cells. Then there is the inner shell surrounding the spherical oocyte^7^. The origin of the considered follicular system is still unclear and there are several hypotheses^18–23^ explaining it.

We have previously described an example of very ordered *Ciona intestinalis* follicular epithelium that has the tetrahedral symmetry and consists of 60 cells^12^. However, in ascidian species a wide variety of types of the follicular system is observed in terms of oocyte volume (the sizes of eggs vary from 100 to 500 $\mu m$ in diameter), morphology, and number of follicle cells that form the spherical polygonal packings without holes. Even the SphEMs of one ascidian species can differ significantly in the number of cells. Common for all samples is that the statistical analysis^10,12,17^ of cell valency demonstrates the overwhelming majority of hexavalent cells. This makes the ascidian SphEMs morphologically similar to other two-dimensional spherical structures with a locally periodic order, including spherical colloidal crystals (SCCs), which are self-assembled at the spherical boundary between two liquids^24,25^. In contrast to SphEMs, the order in SCCs is well studied not only with the simplest statistical analysis, but also with modern topological methods^25–28^. Here, applying them for SphEMs, we for the first time reveal in the cellular structures the so-called extended topological defects (scars and pleats) typical of SCCs^25,29^ and representing linear sequences of alternating cells with 5 and 7 nearest neighbors.

As known, the triangulation of any surface topologically equivalent to sphere must include vertices whose valence differs from 6. In the simplest case, there are 12 such vertices and they are common not for 6, but for 5 triangles. Many natural objects are arranged in such way, for example, some fullerenes and viral shells. However, as it was discovered about 15 years ago^25^ and became one of the foundations of a new science named spherical crystallography, self-organization of many other 2D spherical objects (occurring due to minimization of their free energy) leads to the formation of scars and pleats. According to Ref. 25,such linear defects appear in shells containing about 400 or more structural elements. Similar packaging features arise not only in various coverings of curved surfaces^24,30–32^, but are also closely related to information transmission problems^30,33^, coding theory^30,34^, and other important mathematical problems^35,36^.

In this work we analyze in detail the types of defects detected and the defectiveness level of SphEMs in various species of ascidians. Surprisingly, our study of 140 SphEMs from 8 ascidian species showed that pleats, scars and previously unknown nonlinear defects appear in the spherical monolayer even when the total number of cells forming the follicular system is significantly less than the threshold (for previously known spherical structures) value of ~400. In order to explain this and other topological differences between SphEMs and SCCs we, first, propose a new model for the self-assembly of spherical 2D packing that takes into account both the different effective radii of the interacting particles and their rigidity. The model generalizing the problems of Thomson and Tammes^35,36^ leads to the spherical packings with the topological features similar to those observed in the epithelial monolayers covering the eggs of ascidians. Second, we perform a detailed comparative analysis and topological classification of defects in the considered 140 SphEMs. The defectiveness of the order formed from cells of different sizes, is compared with that observed in SCCs^24,25,37^, which consist of roughly equivalent colloidal particles. It turns out that the size dispersion of particles forming a spherical packing can lead to the formation of extended topological defects in the structures with a particle number *N* significantly less than necessary for the formation of similar defects in patterns constructed from identical units.

## Results

### Model of cellular order in spherical epithelial monolayers

Note that repulsion of particles retained in any finite region (including a sphere surface) leads to their ordering, so this process in various systems can be simulated in a similar way. The self-assembly of the particles retained on a sphere can be modeled with different repulsive pair potentials in the form $1/{r}_{ij}^{\alpha }$, where *r*_*ij*_ is the distance between particles with numbers *i* and *j*, $\alpha \ge 1$ is a parameter^12,35–38^. Spherical colloidal crystals (SCCs) are formed by roughly equivalent colloidal particles located at the spherical boundary between two liquids^24^. These particles are usually charged, so their spherical order is frequently modeled as an equilibrium configuration of equivalent charges retained on the spherical surface^25,39,40^. To obtain such a configuration, initially the particles are randomly distributed on a spherical surface, and then the repulsion energy is minimized.

Nevertheless, our analysis shows that the structures obtained in this way turn out to be significantly less defective than the SphEMs of ascidian eggs. For example, among these SphEMs, monolayers of *Ciona intestinalis* containing the smallest numbers (the average value <*N* > ≈ 94) of cells are more regular but about half of the one-sided images obtained from this species contains 7-valent cells. However, minimization of the repulsion energy (with $\alpha =1$) of the same numbers of identical particles never leads to such a structural feature. The resulting equilibrium structures always contain exactly twelve 5-valent nodes and have no 7-valent ones. (Recall that according to Euler’s theorem^30^, if the triangulation of a sphere contains only 5, 6, and 7-valent nodes, then the number of 5-valent nodes should be more than the number of 7-valent ones by 12.)

**Figure 1:**
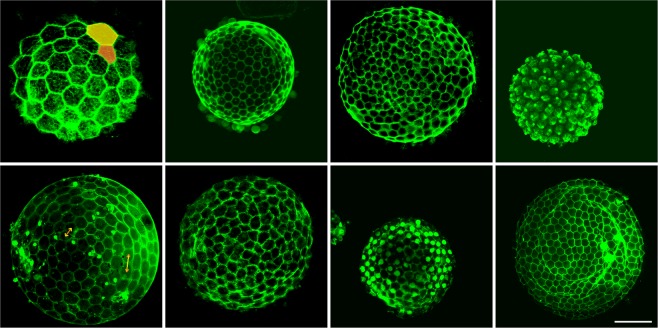
Topological organization of epithelial follicular cells in eight ascidian species (respectively from left upper panel to right lower panel): *Ciona intestinalis*, *Styela clava*, *Ascidia mentula*, *Molgula citrina*, *Ascidiella aspersa*, *Phallusia mammillata*, *Styela plicata*, *Molgula sp*.). Cell membranes were labeled with FITC-WGA in paraformaldehyde fixed oocytes (for more detail, see Methods). All these epithelia are exclusively organized with hexagons, pentagons and heptagons. In the upper left panel, the areas of cells highlighted with yellow and orange differ approximately three times. In the lower left panel, the orange and yellow arrows show the difference (almost twice) in the distances between the centers of neighboring cells. Scale bar (common for all images in the figure): 70 *μm*.

Figure 1 shows spherical epithelial monolayers which form the follicular system of eight ascidian species. One can see that most of the cells in all the panels are hexagonal in shape and more or less probably (depending on the sample) form regions of locally periodic order. It is important to stress that in some monolayers the cell size can vary quite significantly. One can find a pair of cells, the areas of which differ by 3 times, while the distances between the geometric centers of neighboring cells can differ up to 2 times. As we demonstrate below, this geometrical heterogeneity of cells leads to greater defectiveness of the cell monolayers compared to SCCs, the colloidal particles in which are significantly less different in size.

**Figure 2:**
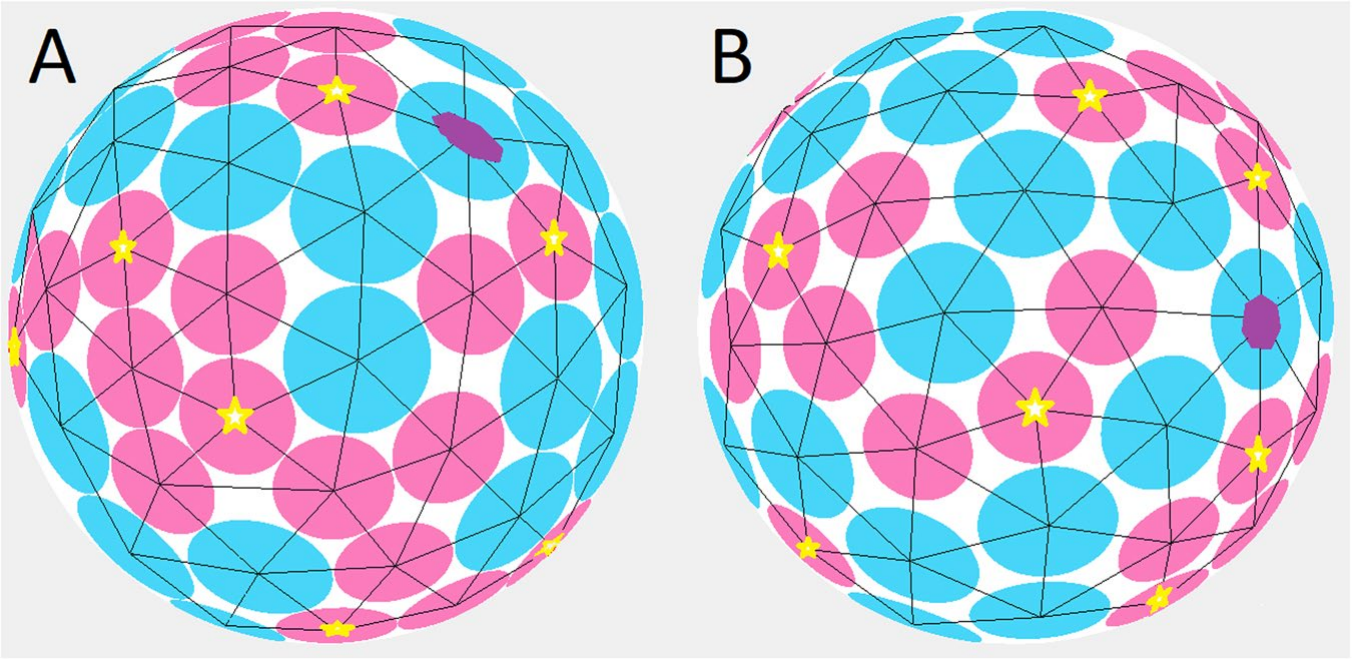
Two model spherical structures decorated with 35 large and 36 small caps, shown in blue and pink, respectively. The blue caps have 20% larger diameter (and ${{\rm{s}}}_{{\rm{i}}}$ value) than the pink. Note that in the left structure (for which *α* = 300) in contrast to the right one (for which *α* = 12), the caps almost touch each other. Caps with 5 and 7 neighbors are marked with small yellow stars and purple heptagons, respectively.

To construct the simplest model allowing us to take the geometrical heterogeneity of cells into account we reconsider the Lennard-Jones energy^41^, which (in the original form) reads

1 $$U=\mathop{\sum }\limits_{i > j}^{N}\left[{\left(\frac{\rho }{{r}_{ij}}\right)}^{12}-2{\left(\frac{\rho }{{r}_{ij}}\right)}^{6}\right],$$

where $\rho$ is the distance at which two-particles interaction reaches its minimum, ${r}_{ij}$ is the distance between the *i*-th and *j*-th particles, *N* is the number of particles. The first term in Eq. 1 describes the contact repulsion, and the second one is responsible for the particle attraction at long distances. Since in our model the particles can be of different size, we replace $\rho$ with the sum of effective radii ${s}_{i}$ and ${s}_{j}$ of interacting particles: *ρ* = *s*_*i*_ + *s*_*j*_. If the particles are retained in a closed space or, in particular, on the surface of a finite sphere, then, in the simplest model, the second term in Eq. 1 can be omitted. Thus, we come to the following interaction energy:

2 $$U=\mathop{\sum }\limits_{i > j}^{N}\left[{\left(\frac{{s}_{i}+{s}_{j}}{{r}_{ij}}\right)}^{\alpha }\right],$$

where *α* = 12 provided we start from Eq. 1. Note, however, that the value of α can vary; the higher this value, the more rigid the structural units become, turning at *α* → ∞ into incompressible spherical caps of fixed sizes, which, unlike the Tammes problem^36^, are not constant but different and proportional to the effective radii ${s}_{i}$ (see Fig. 2). Note also that for the coordinates of the caps’ centers obtained by minimizing the energy (Eq. 2), the absolute values of ${s}_{i}$ are unimportant (as well as the absolute value of the radius of the sphere on which the particles are held). Multiplication of all ${s}_{i}$ by the constant *β* leads only to multiplication of the energy (Eq. 2) as a whole by *β*^*α*^. Since only the ratios between the ${s}_{i}$ values are important, it is convenient to normalize these coefficients so that their average value <${s}_{i}$> becomes 1.

Note that SphEMs of ascidian eggs are non-proliferative, so the variation in the cell size should be random (unlike the case of proliferative cell monolayers, where the cell size is significantly affected by its life cycle phase). Therefore, below we approximate the spread of the cell sizes with Gaussian distribution of random values:

3 $$p({s}_{i})=C\cdot \exp \left(-{\frac{({s}_{i}-1)}{2{\sigma }^{2}}}^{2}\right),$$

where $C=\frac{1}{\sigma \sqrt{2\pi }}$ is the normalization coefficient; $\sigma$ is the mean square deviation of effective radii; *p(s)* is the probability of finding a cell with an effective radius *s* in the model spherical structure; expected value of ${s}_{i}$ is equal to 1. Thus, when modeling SphEMs in the next section, we first randomly and evenly distribute a given number of particles *N* over the spherical surface, specifying their effective radii according to Eq. 3. After that we minimize the energy (Eq. 2) with the ordinary gradient descent method (see more detail in Methods).

### Defects in spherical epithelial monolayers and their modeling

**Figure 3:**
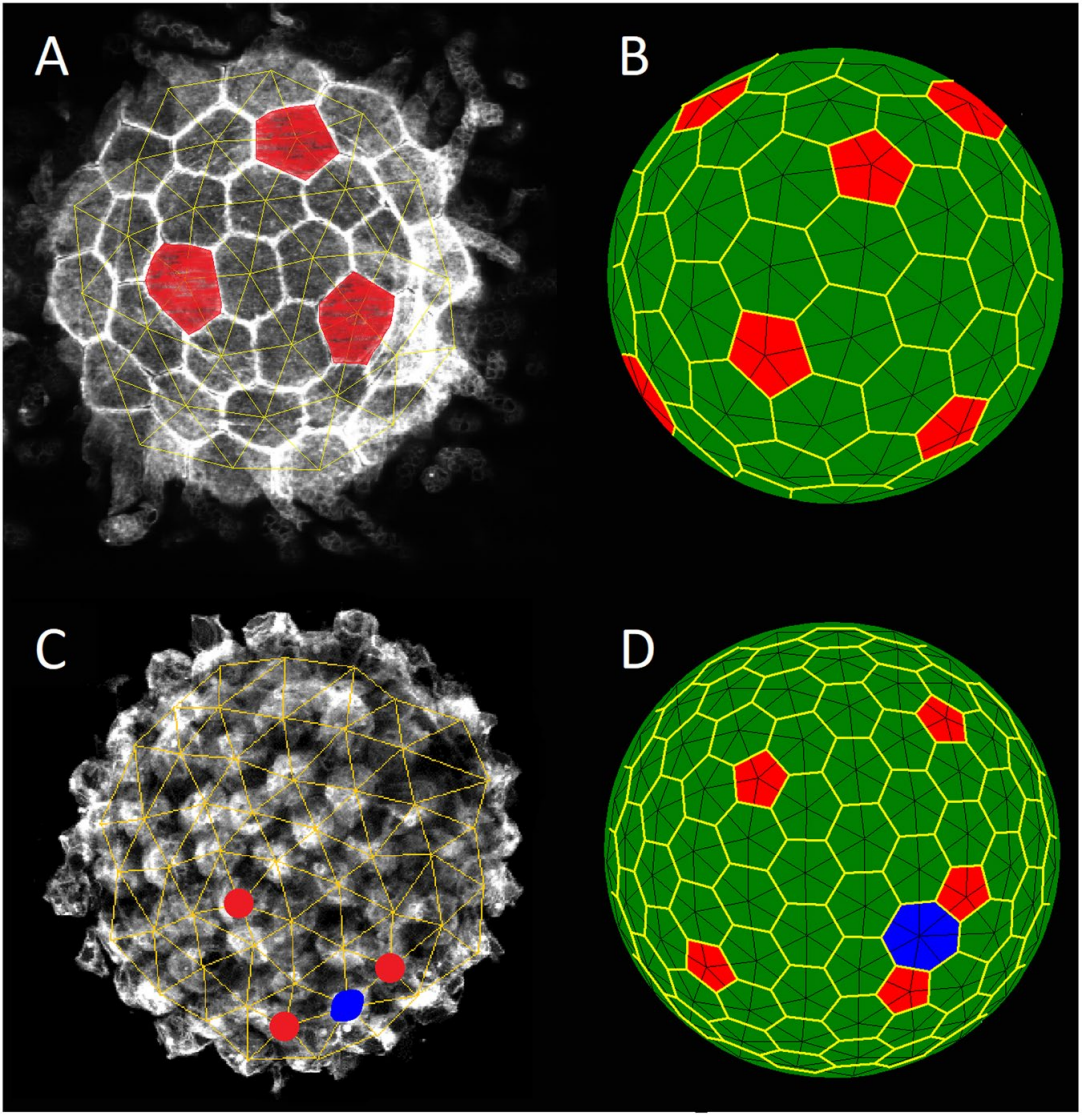
The most topologically regular (**A,B)** and slightly defective (**C,D)** hexagonal spherical orders. (**A,C)** Epithelial monolayers of *Ciona intestinalis* and *Molgula citrina*. (**B,D)** The structures obtained in the frames of the proposed model. Cells as well as polygons of Voronoi tessellation^42^ having 5 and 7 neighbors are marked in red and blue, respectively. In the bottom of the panels (**C,D)**, the simplest scar consisting of alternating 7-valent and two 5-valent cells is highlighted.

In total, we have analyzed 140 examples of SphEMs: *Ciona intestinalis*— 21, *Molgula citrina* — 25, *Ascidiella aspersa* — 36, *Styela clava* — 14, *Styela plicata* — 8*, Ascidia mentula* — 11, *Molgula sp*. — 7. The most ordered SphEMs were observed in the species *Ciona intestinalis* and *Molgula citrina*. The analyzed monolayers included relatively small numbers (58 ÷ 130 and 60 ÷ 210, respectively) of cells with rather close sizes. About 48% of *Ciona intestinalis* and 8% of *Molgula citrina* one-side images had no extended topological defects (ETDs) at all (see the ETD definition and some properties in Methods), like the *Ciona* image shown in Fig. 3A. In Fig. 3B the latter sample was modeled by the packing with *N* = 79, *σ* = 0.045 and *α* = 12. This packing contains exactly 12 cells with 5 nearest neighbors (this relation common for the most ordered packings is caused by spherical topology). The pentagonal cells of the Voronoi tessellation^42^ in the model structure are located at different distances from each other, as well as the pentagonal cells in the epithelial monolayer shown in Fig. 3A.

We emphasize that the above choice of *σ* and *α* values is not unique. If $N\lesssim 300$, analogous most regular model spherical structures may be obtained provided ${\rm{\sigma }} < 0.065$, *α*$\gtrsim$1. At fixed *N* value, with decreasing *σ* and *α*, the probability to obtain such a regular structure increases. Accordingly, the probability to obtain a defective structure with the simplest ETD — 7-valent cell surrounded by two five-valent ones — rises together with α and *N*. In the considered two types of SphEMs these simplest scars are often observed. Such an example of *Molgula citrina* is shown in Fig. 3C. This SphEM was modeled with the spherical structure (see Fig. 3D), where *N* = 172, *σ* = 0.045, *α* = 12. It is interesting that in the model structure the particle with 7 neighbors is surrounded with a larger Voronoi cell. The SphEM cell with 7 neighbors (in Fig. 3C) also has slightly larger area. Nevertheless, larger area does not always correspond to a particle with a larger number of neighbors, but this trend exists.

Different defectiveness of the structures presented in the first and second lines of Fig. 3 is obvious. But to discuss quantitatively more defective monolayers, we need to introduce a topological measure of disorder in spherical structures. In all the most topologically regular structures there are exactly twelve 5-fold point disclinations with a total positive topological charge equal to +12 (see more detail in Methods). There are no defects with negative topological charge in them; therefore, the total negative topological charge is zero in this case. Since the total charge of all defects (both positive and negative) is always preserved, we define the degree of topological defectiveness as the total negative charge of all topological defects ${Q}_{-}=\sum _{i\ge 7}({N}_{i}\cdot (i-6)),$ where *N*_*i*_ is the number of cells (particles) having *i* neighbors, (*i − 6*) is the topological charge of each cell or particle. Along with *Q*_*-*_ we introduce the relative topological defectiveness *Q*_*-*_ */ N*, where *N* is the total number of cells. Such definitions allow us to compare quantitatively the defectiveness of epithelial structures, photographed on one side only (see Methods).

**Figure 4:**
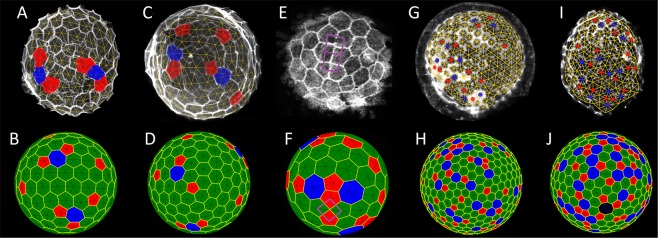
Complex defects in various epithelial monolayers: (**A,C)** *Ascidiella aspersa*, (**E)** *Ciona intestinalis*, (**G)** *Styela plicata***, (I)** *Phallusia mammillata*. (**B,D,F,H,J)** Model structures with defects similar to those observed in SphEMs shown in the upper panels. Cells as well as polygons of Voronoi tessellation with 5 and 7 neighbors are marked in red and blue, respectively. (**A,B)** The SphEM and the model structure with two simplest ETDs. (**C,D)** Simplest dislocations, which consist of neighboring 5-valent and 7-valent cells. (**E,F)** The areas with square order (and ambiguous triangulation) are highlighted with purple squares. (**G,H)** Defective locally hexagonal order with relatively large number of defects. For the visible region of SphEM *Q*_*-*_ = 8, whereas for that of the model structure *Q*_*-*_ = 22. These two latter figures are interesting, because among other defects one can see non-linear complex ETDs. (**I,J**) A highly defective order, where the hexagonal areas become small. In the visible region of SphEM *Q*_-_ = 12, whereas in the one of the model structure *Q*_*-*_ = 27. In panel **(J)** the cell with 8 neighbors is highlighted with dark blue.

Consistent with our model, due to the small number and close size of cells, samples of *Ciona intestinalis* are the least defective monolayers, and about half of them have degree of defectiveness equal to zero (see Fig. 3A). The prevailing majority of the rest SphEMs of this species have 1–2 simplest ETDs. Quite often, simplest scars are formed in *Ascidiella aspersa* structures (see a typical representative in Fig. 4A), which corresponds to defectiveness *Q*_*-*_ (of hemispheres) from 2 to 6. Along with the scars, dislocations may appear in the SphEMs with this defectiveness’ range (see Fig. 4C). The model structures for the *Ascidiella aspersa* SphEMs are shown in Fig. 4B,D.

Even if all particles have the same size (*σ* → 0) and *N* is sufficiently large, ETDs can occur at any value of *α*. However, the observed SphEMs, on average, are more defective than the model structures with the same *N* and *σ* → 0. Size dispersion is needed; in the framework of the proposed model, a formation of structures with several small ETDs (similar to that shown in Fig. 4B) is unlikely at very small *σ*. When *α* = 12 and *N* ~ 150, the highest probability to obtain this-type structure occurs in the range 0.045 <*σ* <0.11. In particular, the structure shown in Fig. 4B, which models one of the *Ascidiella aspersa* samples, is characterized with *α* = 12, *N* = 145, *σ* = 0.1.

Frequently occurring in SphEMs dislocations (see the second sample of *Ascidiella aspersa*, where *N* ~ 195 cells, in Fig. 4C) are unlikely in the model structures with a small difference in particle sizes and *N* <400. When *α* = 12 and *N* ~ 200, the highest probability to obtain a model structure similar to the SphEM shown in Fig. 4C takes place in the range 0.1 <*σ* <0.14. At larger *σ*, simple dislocations turn into longer pleats or cease to be linear defects. Note that the structure shown in Fig. 4D was obtained at the following parameters: *α* = 12, *N* = 195, *σ* = 0.1.

Another surprising defect occurring (although extremely rare) in SphEM is a local area of the square order. The *Ciona intestinalis* sample (N~85) with such defect is shown in Fig. 4E. In the defective area (marked in purple) four different cells have a common contact, which also leads to the ambiguity of the triangulation. The analogous defective area is present in the model structure shown in Fig. 4F; here *N* = 85, *α* = 12 and *σ* = 0.15. Our simulations demonstrate that square-order areas can result from different model parameters: at small α and large spread of the particle sizes (this case corresponds to the structure shown in Fig. 4F) or at constant particle size, but *α* → ∞. Earlier, the emergence of square-order regions was discussed^43^ for spherical model structures consisting of particles of the same size and resulting from the original Lennard-Jones energy (Eq. 1); see also ref. ^44^.

Quite often, more complex, non-linear defects arise in several types of SphEMs. About 70 monolayers studied contained these defects, most often they are found in *Styela plicata, Phallusia mammillata*, and *Molgula sp*. The defectiveness *Q*_*-*_ of the visible hemisphere of monolayers containing non-linear defects is usually higher than 9. The *Styela plicata* sample (*N*~453) with such defect is shown in Fig. 4G. The analogous defective area is observed in the model structure with *N* = 453, *α* = 12, *σ* = 0.18 shown in Fig. 4H. Unlike linear scars and pleats, these ETDs can have a topological charge greater than 1, since several scars and pleats “stick” together into a complex large defect. Such defects are also characterized by significant differences in the size of the cells (or the distances between them) that compose the defect. The greater the number of cells in SphEM is, the more often the non-linear defects occur. In the proposed model, at *α* = 12 these defects begin to occur regularly if *N*> 200 and *σ*> 0.15.

The most defective epithelial monolayer samples, where the area of the defective order is comparable to or greater than the area of the hexagonal order, are found only in three species: *Styela plicata*, *Phallusia mammillata* and *Molgula sp*. It is difficult even to identify separate ETD in these samples. Often, the local regions of hexagonal order are surrounded by a defect order, and the hexagonal order ceases to be simply connected. The defectiveness of the visible hemisphere of such monolayers is of the order of several tens. In such samples, in addition to cells with 5, 6, and 7 neighbors, cells with 8 neighbors are also quite common; however the cells with a larger number of neighbors are very rare. The *Phallusia mammillata* sample (*N* ~ 345) with highly defective order is shown in Fig. 4I. The analogous defective order is observed in the model structure (Fig. 4J), where *N* = 345, *α* = 12, *σ* = 0.26. Also note that the cell with 8 neighbors is present in this packing; a similar defect was found in 16 samples of epithelial monolayers of the discussed species.

## Discussion

**Figure 5:**
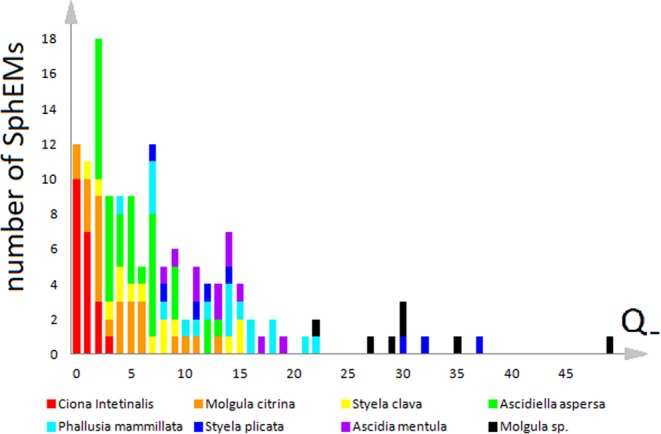
Number of samples with certain topological defectiveness *Q*_*-*_ as a function of *Q*_*-*_ value for eight ascidian species. The height of the colored rectangles shows the number of monolayers of given species that have given defectiveness. Rectangles corresponding to *Ciona intestinalis, Molgula citrina, Styela clava, Ascidiella aspersa, Phallusia mammillata, Styela plicata, Ascidia mentula and Molgula sp*. are shown in red, orange, yellow, green, blue, dark blue, purple and black, respectively.

After the examination of the most typical complex topological defects found in various SphEMs we can proceed to comparative analysis of SphEMs from different species and discussion of the regularities obtained. Figure 5 shows the number of samples with certain topological defectiveness *Q*_*-*_ as a function of *Q*_*-*_ value for eight ascidian species. Since we were able to obtain one-side images only, *Q*_*-*_ functions were obtained for visible hemisphere (see Methods).

SphEMs of *Styela plicata* and *Molgula sp*. are the most defective; their diagrams continue into the region where *Q*_*-*_> 20. In such samples the defects are so complex and extended, that they occupy the main part of visible hemisphere. In some SphEMs, the total topological charge of such a very large defect can be greater than +1, and even there exist SphEMs, where not defects are surrounded by hexagonal order, but small areas of hexagonal order are surrounded by a large defective region. This situation arises in all samples of the *Molgula sp*. type, which have an average topological defectiveness *Q*_*-*_ (corresponding to the visible hemisphere) equal to about 131, which is about 35 times greater than that of *Ciona* *Intestinalis* SphEMs (where *Q*_*-*_ $\approx 3.9$) and about 6 times more than that of *Molgula citrina* and *Ascidiella aspersa*. This essential difference can be explained by the fact that the *Molgula sp*. monolayers (containing ~500 cells on the visible part of image) are not entirely spherical, but rather folded. If the topological defectiveness *Q*_*-*_ is normalized to the number of cells *N* (the both quantities are calculated for the visible part of the sphere) then the relative defectivenesses range from $\approx$0.04 for *Ciona intestinalis to* $\approx$0.18 for *Styela plicata* (see the last column of the Table 1). A total comparative analysis of structural features found in all investigated SphEMs is presented in Table 1.

**Table 1:** Comparative analysis of different monolayers. The lines are ordered according to the relative topological defectiveness of monolayers (see the last column). The value in triangular brackets is the average over all the studied samples.

Species	Average size of eggs, μm	*N* range (Number of cells)	Number of investigated monolayers	<*N*>	<*Q*_*-*_>	*Q*_*-*_ range	*<Q*_*-*_*>/ <N>*
*Ciona intestinalis*	217.6	60–132	21	94	3.9	0–12.8	0.04
*Styela clava*	193.9	269–688	14	441	26.4	6.5–54.5	0.06
*Ascidia Mentula*	236.1	411–931	11	615	51.5	31.5–73.4	0.084
*Ascidiella aspersa*	227.7	112–455	36	222.7	25	8.5–55.4	0.11
*Molgula citrina*	154.2	52–300	25	179.8	21	0–53	0.117
*Molgula sp*.	236.7	411–1467	7	947	131.5	74.2–237.8	0.139
*Phallusia mammillata*	201.9	181–547	18	353	58.4	14.3–97.1	0.165
*Styela plicata*	183	189–616	8	404	73.3	35.7–159.5	0.18

In particular, <*N*> and <*Q*_*-*_> are the average number of cells and the average topological defectiveness for the considered species, respectively.

Summarizing the results of this paper, one can conclude that different defects in various epithelial structures are mainly induced and statistically controlled by two factors. The first of them is topological limitations imposed by the spherical or even more complex curved surfaces, on which the epithelial monolayers are formed. This factor (associated with the Gaussian curvature of surface) also induces topological defects in various physical systems formed from equivalent structural units. The second determinant found characterizes the heterogeneity of the cell sizes and can be described in terms of the corresponding distribution function. As is shown, the second factor, which is absent in known physical systems, increases the topological defectiveness of SphEMs with respect to packings composed from the same number of equivalent structural units. Namely, due to the heterogeneity of cells, the defects typical of the spherical packing containing about or more than 400 colloidal particles^25^, appear in SphEMs formed from essentially smaller number of cells. Thus, the results obtained in the frame of our theoretical model match with the experimental data obtained by means of confocal microscopy (see Figs. 3, 4 and Methods).

Besides these two factors, the effective stiffness of cells (the parameter $\alpha$ in Eq. 2) can also to some extent affect the order and disorder in the considered monolayers. Similarly to the two-minimum Lennard-Jones-Gauss potential^45^ and the step-like square-shoulder one^46^, the proposed energy (Eq. 2), which generalize and unify Thomson^35^ and Tammes^36^ problems, may be useful for modeling various soft matter systems.

It is well known that even a small number of certain defects can change greatly the physical properties of abiotic structures. For example, topological defects can selectively trigger processes of molecular self-assembly in liquid crystals^47^ or distinguish superconductors of the first and second orders^48^. By analogy, the defects we have observed in ascidian epithelia could also play a key role for their biological properties, just as nematic-like defects in Madin-Darby Canine Kidney (MDCK) cell monolayers govern cell death and extrusion^49^. However, the latter comet-like dynamic peculiarities in the cells’ velocity field, only resemble the static topological defects in nematic liquid crystals, which are certain static changes in the director’s orientation. Here we studied the static defects in SphEM structures rather than the features of velocity field that characterizes the epithelium cells. Therefore, the defects we found in SphEMs are really analogous with those well known for various abiotic systems including discussed SCCs. In this context, our study is extremely interesting for new crystallography^25–27,29,39^ of curved 2D crystals since SphEMs along with the defects typical of single-layer planar structures contain the curvature-induced ones.

In addition to using the biological system for generalizing the problems of curved spherical crystals, we demonstrated that various defects in epithelial structures manifest themselves as an adaptive plastic response to the limitations imposed by spherical packing of heterogeneous cells. Indeed, some degree of imperfection can help, for example, to maintain the function of impermeability and the reparative potential of the epithelium throughout the morphogenetic process, leading to terminal differentiation of oocytes. Note that *Ciona intestinalis* follicular cells are subjected to massive apoptosis during early development^12,50^. Similar to the determining influence of nematic defects on MDCK cells apoptosis^49^, the topological defects found in *Ciona intestinalis* could also have a key role in initiating the apoptotic process necessary for ascidian development. Analogous assumption could also be made for morphogenetically active embryonic epithelia. For example, Toyama *et al*.^51^ showed that during dorsal closure, delamination of the apoptosing amnioserosa cells produces forces that concurrently facilitate cell extrusion and promote dorsal closure in the *Drosophila* embryo. In a morphologically similar scenario, mouse neural tube closure requires apoptosis^52^.

The role of the investigated topological defects in initiating the apoptotic process will be tested in our future study by analyzing the position of the first apoptotic cells in relation to the position of defects in non-active follicular monolayer of ascidians and/or in morphologically active *Drosophila* embryonic epithelial cells.

## Methods

### Labeling and fluorescence microscopy

Just after gonads dissection, oocytes were collected and fixed for 20 minutes with 3.7% formaldehyde in filtered seawater. The oocytes were permeabilized with 0.2% Triton in phosphate-buffered saline (PBS), washed once in PBS and processed for cell contour labeling with Wheat Germ Agglutinin (WGA). Oocytes were then washed three times in PBS, once in Tris-buffered saline (TBS), rinsed in distilled water and mounted in Mowiol (Sigma). In all ascidian species the follicular cells possess an apical refringent body whose function is unknown. In all the species studied, except *Molgula citrina* and *Styela plicata*, it is possible to eliminate this structure by mechanical agitation, which facilitates the accessibility of the WGA to the basal contour of the cells. In the case of *Molgula citrina* and *Styela plicata*, mechanical agitation does not eliminate the refringent body and the WGA concentrates around this structure at the top of the cell to the detriment of its basal contour. Thus for these two species, the usage of WGA allows us to label the tops of the cells. The slides were analyzed with a Leica SPE laser confocal microscope (Montpellier RIO Imaging platform, France).

### Image analysis

After determining the geometric centers of the cells, triangulation was performed by the Delaunay method^53^. In the cases of *Molgula citrina* and *Styela plicata*, the centers of the labeled cellular tops were used as the nodes of the triangulation. Then, around the SphEM images, a maximally large circle was chosen with such a radius that only the centers of cells that are completely visible are located inside the circle; an additional condition was that the nearest neighbors of cells inside the circular boundary were at least partially visible allowing one to determine the valence of the inner cells. The approximate total number of cells in the entire sphere was calculated as

4 $$N\approx \frac{2n}{1-\sqrt{1-{\left(\frac{r}{R}\right)}^{2}}},$$

where *n* is the number of cells located in the circle under consideration, *R* is the radius of the considered SphEM sample. For elongated samples, a quarter of the sum of the largest and the smallest visible diameters was taken as the radius. After that, for the cells in the circle one can determine the number of nearest neighbors and calculate the approximate defectiveness *Q*_-_ of the entire sphere as the defectiveness of the selected area multiplied by the ratio *N/n*.

The error in determining the value of *N* is caused by several factors, including the inaccuracy in determining the size of the sphere that corresponds to the epithelial monolayer (arises due to blurring of the image along the boundaries), asphericity of the sample border and inhomogeneity of cell’s distribution over the sample. Application of Eq. 4 to model structures with a known number of particles shows that for constant particles size the error in determining the value of *N* does not exceed 3%, and for particles with different sizes (*σ* ~ 0.2) the error does not exceed 5%. In addition to the above reasons, the error in determining *Q*_*-*_ also strongly depends on inhomogeneity in distribution of defects over the sphere. Therefore, the value of *Q*_*-*_ is determined with a much larger error than the value of *N*.

### Minimization of model energy

We carry out minimization of the energy (Eq. 2) over the coordinates of the particles retained on the sphere surface. In general, gradient descent leads to the nearest energy minimum. So the resulting spherical packings are very sensitive to the initial coordinates and effective radii of caps and the majority of packings obtained with the gradient descent correspond not to global but to local minimа of energy (Eq. 2). As is well-known for the case of $$\alpha $$ = 1 and equivalent particles, if their number *N* increases, then the distances between the energy levels rapidly decrease and the number of possible equilibrium structures grows exponentially^54^. Therefore, at sufficiently large values of *N*, it is practically impossible to get by chance an equilibrium structure corresponding to the global minimum of energy, while the theoretical search for such structures represents a complex mathematical problem^44,55^. As far as we know, this problem was previously discussed only for the case of equivalent particle ($${s}_{i}=1).$$ If $$\alpha $$ = 1 the problem is named after J.J. Thomson^35^, while for the case of $$\alpha \to \infty $$ it is the Tammes problem^36^. However, all SphEMs studied in this work are unique and have various structures. In our opinion, this indicates that these asymmetric heterogeneous packings most likely correspond to numerous local minima of the considered free energy, rather than global ones. In addition, we note that the structures of colloidal crystals also correspond to local minima^29,56^ of the repulsion energy (2), where $$\alpha $$ = 1 and $${s}_{i}=1$$.

Nevertheless, at absence of caps heterogeneity and some very specific small *N* values, the number of minima can be very limited. In the particular case when *N* = 60, 8 ≤ α ≤ 12 and for all caps *s*_*i*_ = 1, regardless of their initial coordinates on the sphere, the minimization of energy (Eq. 2) always results^12^ in the same spherical structure with the symmetry *T*. The location of caps in the structure is quite similar to that of the cells in one of the *Ciona Intestinalis* SphEM samples.

### Topological analysis of locally periodic hexagonal order on a spherical surface

According to the regularity of the defects location and the presence of non-point (extended) defects, two-dimensional ‘crystals’ with a spherical topology can be divided into more and less ordered. The most ordered are viral capsids that satisfy the well-known geometric model of Caspar and Klug^57^ and some fullerenes, for example C_60_. The Gaussian curvature of these shells induces only point topological defects (disclinations) lying at the vertices of a regular icosahedron. These highly ordered shells can be obtained by mapping a flat hexagonal order onto a 3D surface using the icosahedral net.

The most studied examples of less ordered systems are spherical colloidal crystals, which arise at the boundary between two liquids^24,25^ and have a structure with various ETDs^25,28,58–60^ absent in highly ordered systems. In the spherical and other curved colloidal crystals, the Gaussian curvature induces, along with the point disclinations, so-called scars^25,58^ and pleats^28,59,60^. A pleat, like a scar, is a chain of alternating colloidal particles having 5 and 7 neighbors, however, in pleats, unlike scars, the numbers of particles with 5 and 7 neighbors coincide. In all sufficiently large spherical colloidal crystals (with the number of particles $N\gtrsim 400$) the presence of ETDs in the form of scars^25,29,58^ becomes mandatory; in addition, the short pleats (simplest dislocations) can be also observed.

**Figure 6:**
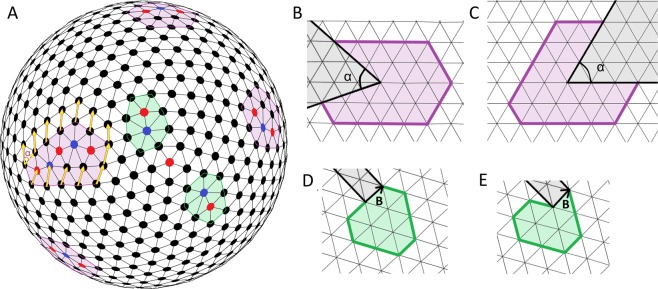
Slightly defective model spherical hexagonal order formed by *N* = 700 repelling particles (*α* = 1). (**A)** Triangulation of spherical model structure. Red and blue circles denote the particles with 5 and 7 nearest neighbors. Extended topological defects (ETDs) (all with the topological charge +1) and dislocations are located within violet pentagons and green hexagons, respectively. The shown dislocations are the simplest and look like a pair of adjacent particles with 5 and 7 neighbors. (**B,C**,**D,E)** The boundaries of the ETD (shown in the left of **A**) and dislocation (shown in the down of **A**) after their transfer onto the planar hexagonal lattice. Both contours after their transfer to the plane become unclosed. The comparison between (**B,C**) and (**D,E**) shows that in contours surrounding both types of defects the breaking point can be chosen arbitrarily. The circulation of a hexagonal translation (the chosen one is shown by the yellow arrows) around the ETD on the sphere, leads to the translation turn on *α=π*/6.

If the curvature radius of a curved crystal is comparable to its size, then the most energetically favorable way to compensate for the curvature is the appearance of topological defects. Some of these defects (an isolated pentagon which is the point disclination and four ETDs with broken hexagonal order inside them) are visible in Fig. 6A. The defects can be surrounded by a pentagonal contour whose sides are parallel to the minimal translations of the hexagonal order^29^. As shown in Fig. 6A the circulation of the hexagonal order translation along the contour surrounding the defect leads to the translation rotation on the angle *α* = *πZ*/3, where *Z* is the topological charge of ETD. For the considered case *α* = *π*/3, and in order to close this contour, a *π*/3 sector should be cut out from the hexagonal lattice (see Fig. 6B,C). Thus such an ETD (with a topological charge +1) arises due to a *π*/3 sector eliminated from the planar hexagonal order. This elimination compensates for a positive Gaussian curvature, and adding the same sector compensates for negative (saddle-like) curvature and creates a defective area with topological charge −1. However, in slightly defective spherical hexagonal order, ETDs with a total negative charge are absent.

Single particles with 5 and 7 neighbors can be also considered as point disclinations, carrying, respectively, topological charges +1 and −1. The topological charge of a particle is a quantity that characterizes how much the nearest order around it is different from the hexagonal one. In this case topological charge is equal to 6 minus the number of neighbors of the particle. In any case, according to the Euler theorem, the total topological charge of all defects on any surface with a spherical topology is 12. Note, however, that the planar order as well as an order on the torus surface is topologically neutral.

In spherical crystal, the total topological charge of dislocations and pleats is absent (see Fig. 6A). The circulation of the hexagonal order translation around the dislocation (or pleat) is zero, but when the surrounding contour is transferred onto a flat hexagonal lattice, the contour becomes broken, and the Burgers vector **B** (see Fig. 6D,E) closes it. Any pleat is topologically equivalent to dislocation and can be also characterized by the Burgers vector.

## Data Availability

Data supporting the findings of this manuscript are available from the corresponding authors upon reasonable request.
